# Treatment of impacted canines with aligners: An alternative and viable option

**DOI:** 10.1002/ccr3.4856

**Published:** 2021-09-22

**Authors:** Gianluca Mampieri, Tommaso Castroflorio, Andrea Conigliaro, Aldo Giancotti

**Affiliations:** ^1^ Department of Clinical Sciences and Translational Medicine University of Rome Tor Vergata Rome Italy; ^2^ Department of Surgical Sciences Dental School University of Torino Turin Italy; ^3^ Orthodontist Private practice Siracusa Siracusa Italy

**Keywords:** aligner treatment, customized treatment, impacted canines

## Abstract

To recover impacted canines without esthetic issues, the aligners can be a resolutive tool allowing by pontics the camouflage of absent canines during orthodontic treatment. Knowledge of biomechanics, correct staging of dental movements, and surgery planification are strategic to achieve a good result.

## Graphical abstract

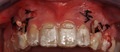

## 1 INTRODUCTION

Impacted cuspids represent an important challenge for orthodontists, particularly when we have to treat patients rejecting fixed and visible appliances. Currently, aligners allow to face several types of malocclusions, but there are few indications for impacted canines, especially without the support of auxiliaries as miniscrews or fixed sectionals.

However, the combined use of aligners and elastics can be effective in the treatment of impacted canines as shown by this clinical report. A 17‐year‐old female patient had upper deciduous canines in the arch and permanently impacted cuspids inside the alveolar bone. After a first phase of treatment aimed at recovering space in the arch, it was planned to surgically expose both canines to pull them directly toward their ideal position without resorting to any external auxiliary other than direct elastic forces from the canines to the aligner.

To achieve the mentioned objectives, without causing esthetic issues, the aligners were modified directly by the orthodontist in order to allow elastic traction of the impacted canines while keeping a pontic to camouflage the absence of the canines in the arch.

The final result supports the idea that, if the position of the impacted canines is favorable, it is possible to recover them by using simple biomechanics with aligners and without auxiliaries.

Impacted teeth can represent one of the most challenging clinical issues for orthodontists. In particular, the management of impacted canines is often complex and shall be associated with a careful physical and radiological examination. According to literature, the inclusion of canines, following third molars, occurs the most frequently, with an incidence ranging from 1% to 5.9%.^1^ As a result, several orthodontic techniques, including specific devices and surgical approaches, have been proposed to move an impacted canine into the arch. Most techniques imply the use of fixed appliances and a number of auxiliaries in order to favor a guided forced eruption in the crestal bone.^2^, ^3^, ^4^, ^5^, ^6^ Yet, in the last few years, a growing demand for orthodontic treatment with removable esthetic appliances as aligners can be noticed, especially for the adult population.

When the Invisalign® system was introduced, it had some limits, such as the inability to control root movement and to move larger teeth over substantial distances.^7^, ^8^ Recent improvements have allowed the use of Invisalign® also in more complex clinical conditions, thanks to innovative material and attachments, more accurate software, the introduction of a new force system, and the more extensive experience gained.^9^, ^10^, ^11^, ^12^, ^13^, ^14^, ^15^ The aim of this work is to share a dedicated procedure for the treatment of impacted cuspids by combining the use of aligners with a conventional forced eruption technique.

## 2 CLINICAL REPORT

### 2.1 Diagnosis

A 17‐year‐old female patient presented with a Class I occlusion, mild overbite, and missing upper cuspids with the permanence of the respective deciduous.

**FIGURE 1:**
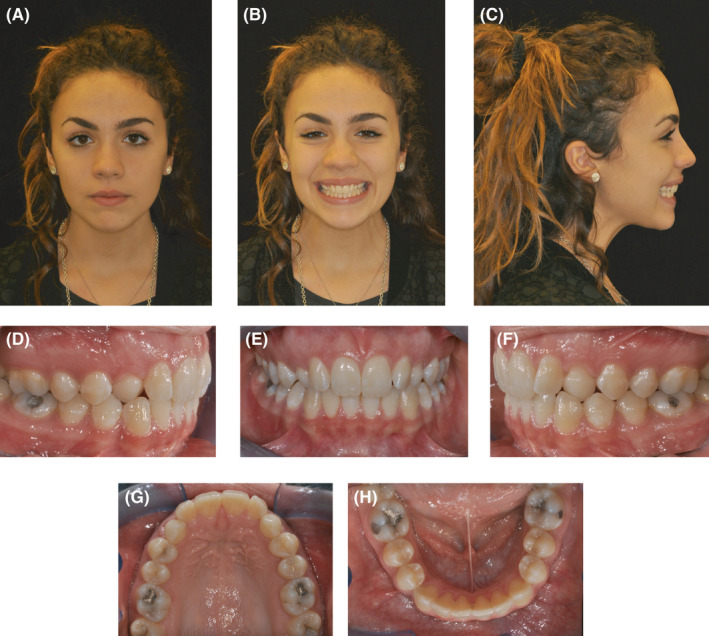
(A‐H) Extra and intraoral pretreatment records

**FIGURE 2:**
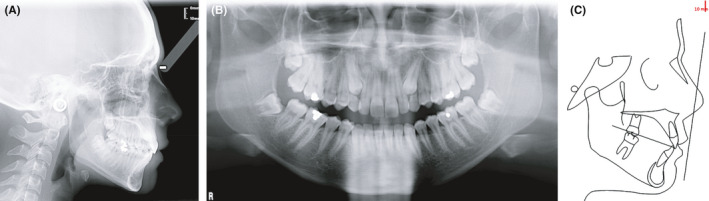
(A) Pretreatment lateral X‐ray. (B) Pretreatment panoramic X‐ray. (C) Initial cephalometric tracing

**FIGURE 3:**
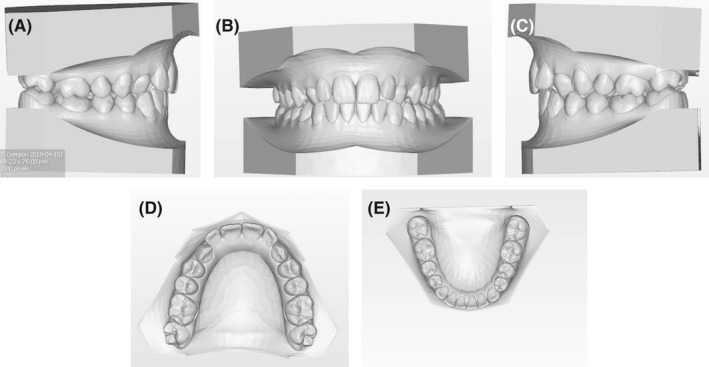
(A‐E) Initial digital casts

The upper and lower incisors were slightly retrusive. The patient's smile was not esthetic due to the presence of deciduous canines in the arch. The maxillary midline was coincident with the face and the mandibular midline. As confirmed by the panoramic x‐ray, the upper canines were impacted. All third molars were developing, and there were no recordable periodontal alterations. (Figures 1A‐H; 2A‐C; 3A‐E).

### 2.2 Treatment objectives

Treatment objectives were to:

1: align and level both arches;

2: gain space in the upper arch for impacted canines;

3: recover impacted canines;

4: refine alignment and occlusion.

### 2.3 Treatment alternatives

Given the clinical conditions revealing that the two impacted cuspids were positioned at the center of the alveolar crest, meanwhile considering the minimal space required for their recovery, several specific devices could have been applied. In literature, there are hundreds of publications reporting several effective methods pursued in order to recover impacted canines.^16^, ^17^, ^18^, ^19^ The majority thereof reports the utilization of full or segmental fixed appliances able to exert rational forces and specific biomechanics.^20^, ^21^, ^22^, ^23^ Additionally, other articles report the use of skeletally anchored devices demonstrating their effectiveness for the treatment of complex canine impaction.^24^, ^25^, ^26^

However, regarding the reported case in this article, two considerations led us to prefer aligner treatment for two main reasons: firstly, the awareness of having to treat two not deeply impacted canines; secondly, the patient's great esthetic expectations. The patient was informed that the treatment could have lasted slightly longer if compared with conventional fixed appliance timing.

### 2.4 Treatment plan

The orthodontic treatment with aligners would include 3 phases:

Phase I: slight expansion of the arches, correction of incisor inclination, and space gain for 1.3 and 2.3;

Phase II: surgical exposure of impacted canines (1.3 and 2.3) following extraction of deciduous canines, and their engagement and traction toward the arch;

Phase III: orthodontic positioning of impacted canines, outreach of the most proper position into the arch, and optimal esthetics.

**FIGURE 4:**
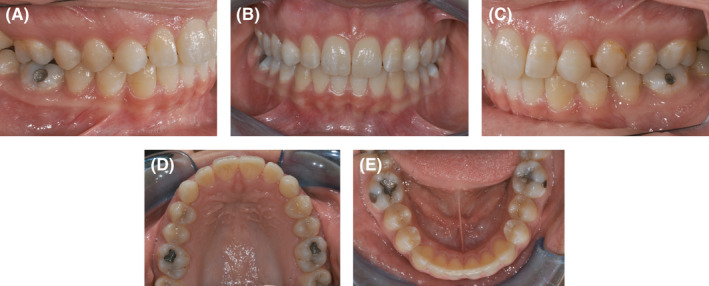
(A‐E) Clinical observation after using 21 aligners

The first phase consisted of 21 aligners to gain enough space for 1.3 and 2.3 by expanding the arch without significant incisor proclination. (Figure 4A‐E).

The second phase followed the extraction of deciduous canines and consisted of 17 aligners. This treatment phase was more challenging because we had to manage the pontics in the aligners to obtain a valid camouflage of the cuspids' absence during the traction of 1.3 and 2.3 and upon the elastic traction to guide the impacted teeth into the arch. The innovative feature of the mentioned treatment phase was the guided eruption of 1.3 and 2.3 without using auxiliaries, but rather aligners only.

The third and last phase was aimed at finishing occlusion and intercuspation, by using 13 aligners.

### 2.5 Clincheck virtual planning

**FIGURE 5:**
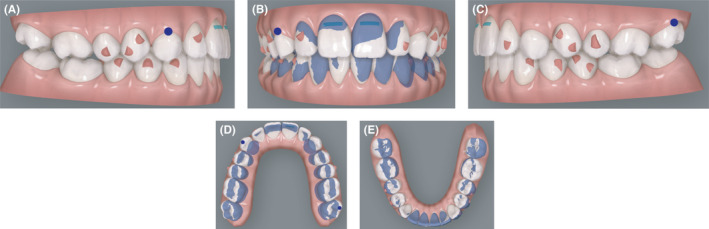
(A‐E) Initial virtual projection

The first treatment phase consisted of 21 aligners in each arch (Figure 5A‐E). The planned objectives were to gain further space for impacted cuspids by means of upper arch expansion and slightly proclining incisors. During this stage, alignment, leveling, and coordination of both arches were also planned. Moreover, upper molars were distally derotated, whereas lower molars and premolars were tipped buccally mostly to perform relative extrusion with a strategic advantage in deep bite correction. Optimized attachments were used for this step.

After having completed the surgical phase, the last three upper aligners were modified directly by clinicians in order to allow the use of intra‐arch elastics and to engage impacted cuspids, and then traction them toward the arch.

**FIGURE 6:**
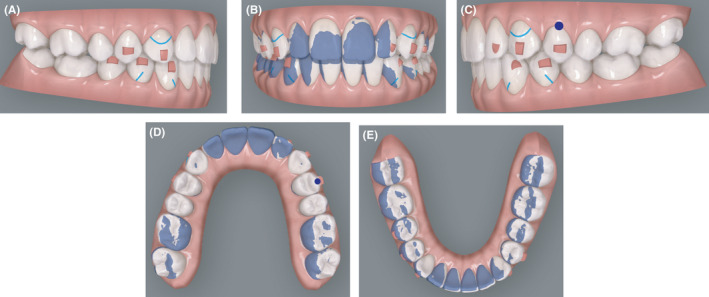
(A‐E) Intermediate virtual projection

The second step aimed at guiding the upper canines in the arch was planned by including 13 aligners (Figure 6A‐E). Because the active extrusion forces applied to the cuspids were exerted by interarch elastics, we planned precision cuts on the aligners in the 1.3 and 2.3 areas. These cuts were utilized to bond esthetic buttons on the surface of 1.3 and 2.3. Hooks were projected in the lower arch corresponding to the 3.3, 3.4, 4.3, and 4.4 areas. This way, it was possible to use triangle elastics from the esthetic buttons on the upper canines to the hooks on the lower aligners.

At this stage, we preferred conventional attachments on lower premolars, as they would guarantee greater stability to the aligner once the elastics were worn by the patient. Moreover, on the upper canines, we prescribed rectangular attachments to enable greater control of the canine tip upon tooth extrusion.

**FIGURE 7:**
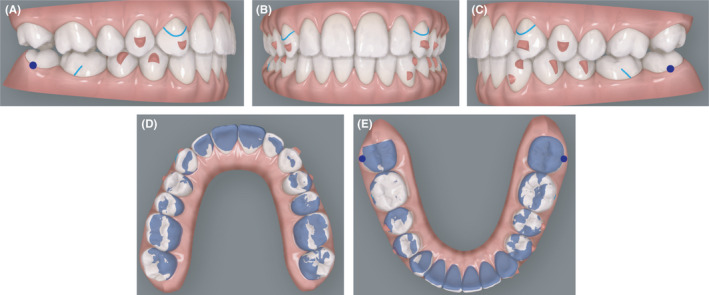
(A‐E) Finishing virtual projection

The finishing phase of treatment consisted of 17 aligners aimed at distally tipping 1.3 and 2.3, thus improving occlusion (Figure 7A‐E).

Optimized extrusion attachments were placed on 1.4, 4.4, 4.5 and 2.4, 3.4, 3.5 in order to reach the best possible arch intercuspation. Moreover, the use of Class II elastics from a metallic button on 1.3 and 2.3, as well as hooks on the aligners in 3.6 and 4.6 was planned in order to achieve a slight extrusion and the distal tip of canines.

### 2.6 Treatment progress

The described innovative treatment was based on a surgical approach combined with the management of cuspid‐guided eruption by using aligners.

**FIGURE 8:**
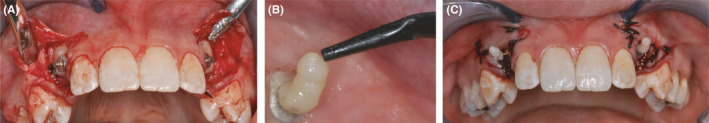
(A‐C) Surgical and bonding phase

Once cuspid space was obtained, the surgical phase was carried out with the preliminary extraction of deciduous canines. Bilateral full‐thickness flaps were raised, and the cortical bone was removed until the partial exposition of the impacted canine cusp. A metal button was sandblasted on its surface and bonded to the exposed canine crown. After applying an adhesive film on the button's surface, a horn‐shaped composite hook with a flow (Core‐× Flow A3, Dentsply) was created starting from the metal button (Figure 8A‐C). The flap was then repositioned and sutured so that the composite hook would be accessible to the patient.

**FIGURE 9:**
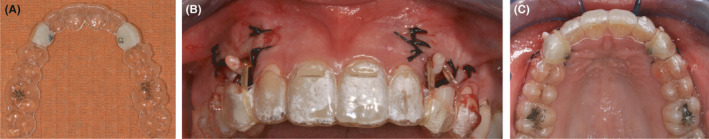
(A‐C) Orthodontic traction of the cuspids

Just 2 days after surgery, the buttons were placed inside pontics, where a hole had been made on the palatal side to enable their exit. They were fixed within the same resin of temporary crowns that had been used to fill the pontics simulating 1.3 and 2.3 crowns. Thus, the patient was instructed to apply intra‐arch elastics (6 mm/180 g) from the composite hook to the second palatal buttons for anchorage (Figure 9A‐C).

It was indicated to wear elastics at least 12 h/day. Thanks to the elastic traction, the permanent canines began to erupt guided by the aligner at the center of the alveolar process. Indeed, every 20 days, this process implied that the resin inside the pontics had to be gradually reduced in order to make space for canine crowns.

**FIGURE 10:**
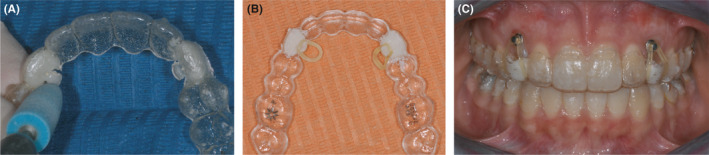
(A‐C) Orthodontic traction from “dovetail” hooks to impacted canines

After 3 months, when the cuspid tips were more superficial, the buttons were replaced, and the composite arm was no longer necessary. Also, the elastics were replaced with shorter ones (4 mm/180 g). Since then, the second button in the aligners was replaced by a "dovetail" hook created on the palatal side corresponding to areas 1.3 and 2.3. The intraoral elastic (4 mm/180 g) was directed from the button on the canine to the “dovetail” hook. (Figure 10A‐C).

During the cuspid traction phase, the pontics allowed to maintain suitable esthetics. The pontics in areas 1.3 and 2.3 were filled with a thin layer of composite (Core‐× Flow A3, Dentsply) on the buccal surface only. This way, there would be enough space to enable forced canine eruption.

**FIGURE 11:**
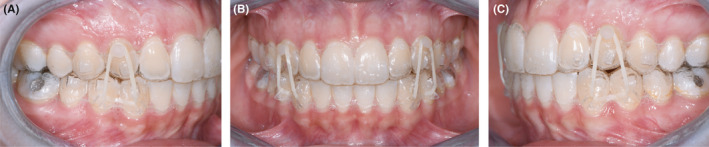
(A‐C) Refinement stage with triangle elastics applied

After only 2 months, both cuspids were in the arch, after which Phase III was launched. Additional aligners were planned to refine occlusion and esthetic details. (Figure 11A‐C).

### 2.7 Treatment results

**FIGURE 12:**
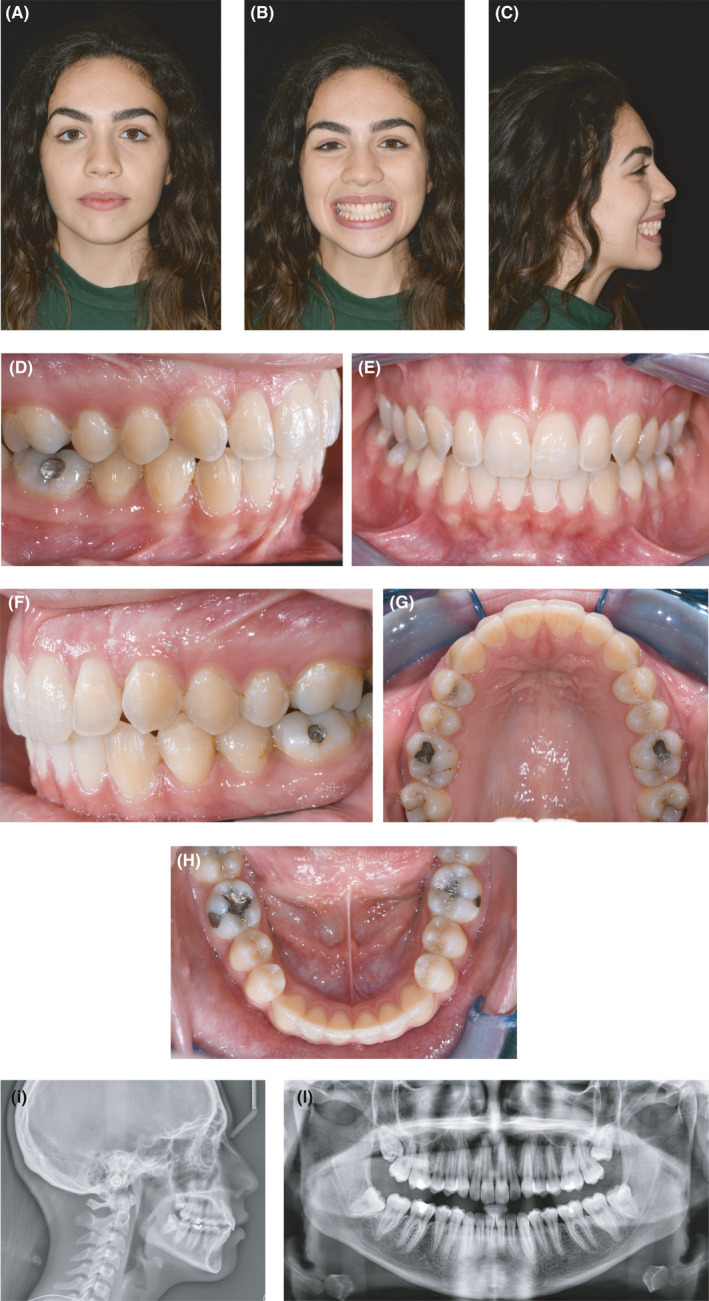
(A‐H) Extra and intraoral posttreatment records. (I) Posttreatment lateral X‐ray. (L) Posttreatment panoramic X‐ray

Final clinical records show good esthetics and functional recovery of upper canines in the arch. The patient's smile considerably improved, and the treatment was completed within 18 months. (Figure 12A‐L).

**FIGURE 13:**
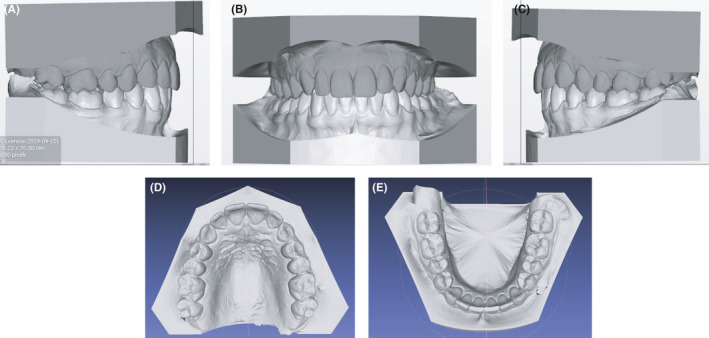
(A‐E) Final digital casts

At the end of treatment, periodontal evaluation showed healthy marginal tissues with pale pink and firm gingivae. Also, the gingival smile line was improved, with the gingival parabolas of the canines at the same height of the central incisors, contributing to the pleasant smile esthetics. (Figure 12) Occlusal results evidenced a proper bilateral Class I relationship and a correct overbite. A mutually protected occlusion with canine guidance during excursive movements was achieved. Proper interdigitation of the upper functional lingual cusps into the fossae of the lower teeth was achieved. (Figure 13) Moreover, upper and lower midlines were facially centered. (Figure 13).

**FIGURE 14:**
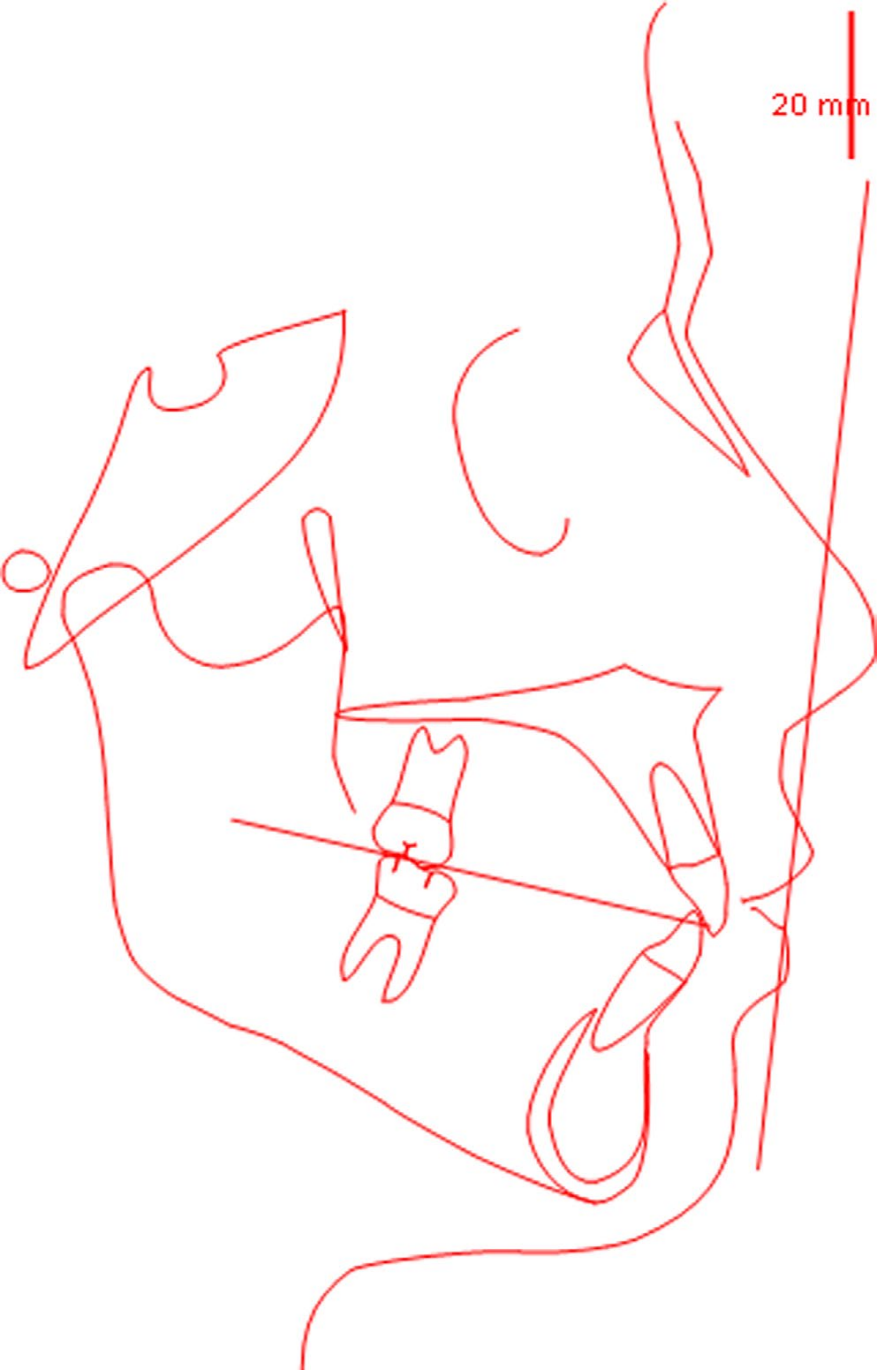
Final cephalometric tracing

**FIGURE 15:**
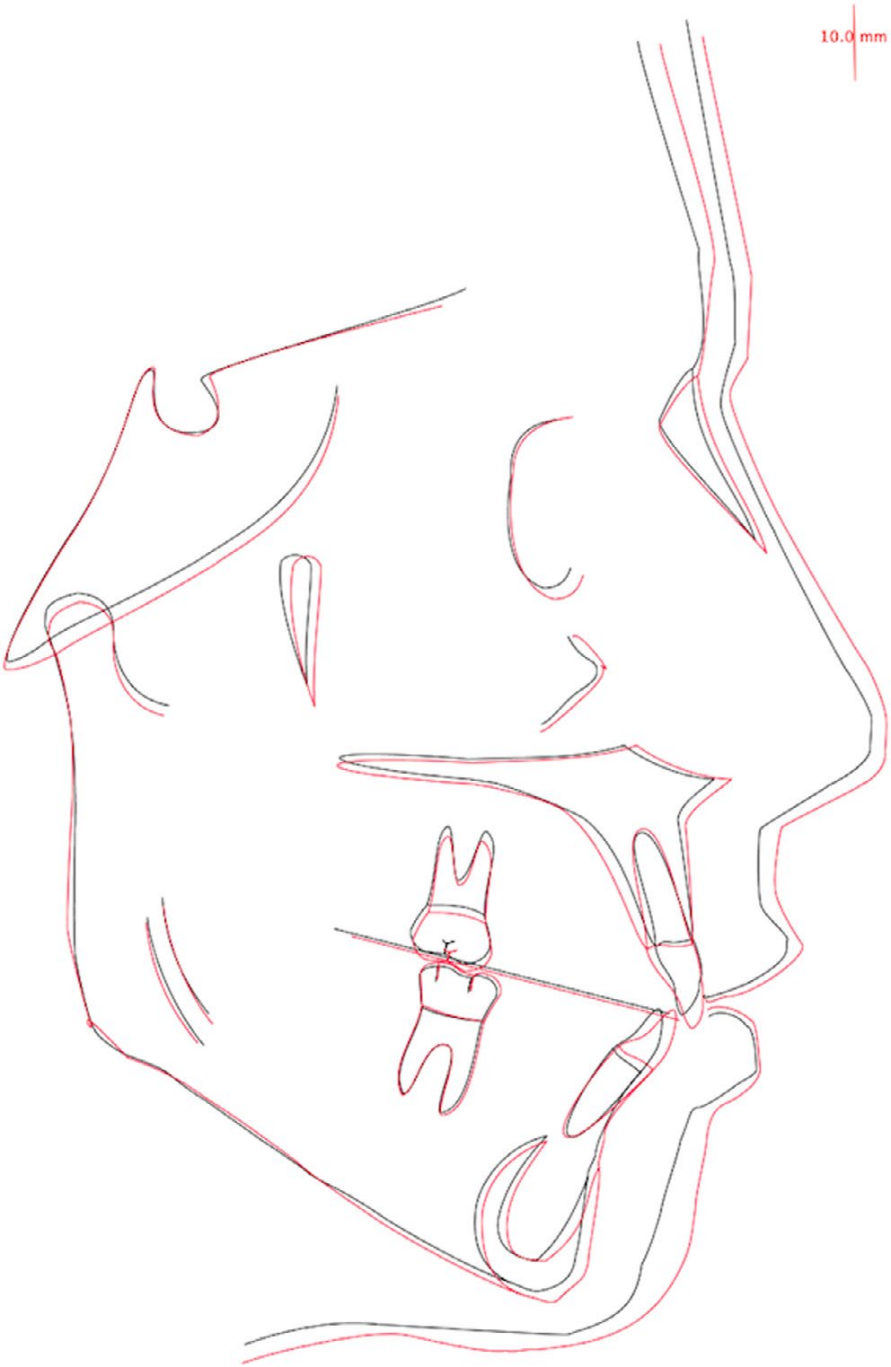
Pre/Post superimposed cephalometric tracings

Final tracing (Figure 14) and overall superimposed cephalometric tracings (initial and final; Figure 15), and their quantitative values underscored the improvement of the front teeth position. Indeed, both upper and lower incisors were slightly proclined, while posterior teeth were maintained in the initial position. (Table 1).

**TABLE 1:** Cephalometric assessment

Cephalometric morphological assessment	mean SD	Pre	Post
Sagittal skeletal relations			
Maxillary position S‐N‐A	82° + 3.5°	85°	85°
Mandibular position S‐N‐PG	80° + 3.5°	80°	81°
Sagittal jaw relation A‐N‐PG	2° + 2.5°	5°	4°
Vertical skeletal relations			
Maxillary inclination S‐N/ANS‐PNS	8° + 3.0°	4°	4°
Mandibular inclination S‐N/GO‐GN	33° + 2.5°	33°	33°
Vertical jaw relation ANS‐PNS/GO‐GN	25° + 6.0°	29°	29°
Dento‐basal relations			
Maxillary incisor inclination 1/ANS‐PNS	110° + 6.0°	99°	109°
Mandibular incisor inclination 1/GO‐GN	94° + 7.0°	93°	97°
Mandibular incisor compensation 1/A‐PG (MM)	2 + 2.0 mm	3 mm	4 mm
Dental relations			
Overjet (MM)	3.5 + 2.5 mm	2 mm	2 mm
Overbite (MM)	2 + 2.5 mm	3 mm	3 mm
Interincisal angle 1/1	132° + 6.0°	139°	125°

At the end of the treatment, dedicated thermoformed retainers (Vivera®) were dispensed to the patient in order to promote the long‐term stability of results.^27^

## 3 DISCUSSION

Nowadays, it is possible to correct an increasing number of malocclusions by using aligners, including impacted canines. Previously, however, impacted canine treatment was considered as being challenging and often requested a hybrid approach featuring both aligners and sectional fixed appliances.^20^, ^21^, ^22^, ^23^ Indeed, the traction phase of impacted canines used to be considered possible only by adopting an auxiliary system as sectional fixed appliances or TADs.^24^, ^25^, ^26^

Our work underscores that, in certain clinical conditions, one can guide impacted canines by using aligners combined with elastic traction.

An accurate preliminary study with CBCT is mandatory to localize the 3D position of impacted canines and to decide how to approach them. If the canine is in a favorable position, at the center of the alveolar crest and not too deep, the intra‐arch elastic system and aligners can be used to guide cuspids into the arch.

The advantages of this treatment are several, including:

1. Good esthetics: we can use aligners with pontics to obscure the absence of the canines; elastics can be worn at home only.

2. Effective management of reaction forces: during the traction of impacted canines with fixed appliances, one of the most difficult aspects is to plan the anchorage and to control reaction forces which often generate undesired asymmetries in the maxillary arch. For this reason, it is advisable to use transpalatal bars, TADs, and full‐size wires, yet several difficulties can arise while using them. With aligners, reaction forces will be released on all upper teeth, producing only a slight intrusion.

3. No emergencies upon traction phase: the complex system to extrude impacted cuspids and relevant forces can often lead to the debonding or detachment of fixed appliances. This problem is overcome with the use of aligners.

Canine‐forced eruption technique with surgery closed approach has been widely described in the literature, but it has been never associated with aligners and with elastics activation system managed directly by patient. In every device described in the literature are present fixed appliance and/or anchorage system that preview an additional surgery phase for insertion of miniscrews or plates.^23^, ^24^, ^25^, ^26^, ^28^ Our system is clearly less invasive and it guarantees esthetic and less stress for the patient who can decide during the day when is the best time to wear elastics for extruding impacted canines.

Yet, there are also some limits in the use of aligners to treat impacted canines. Hence, an accurate preliminary study is crucial, because the indication is strictly dependent on the favorable position of the impacted canines, as previously explained.

## 4 CONCLUSIONS

Current orthodontics increasingly calls for esthetic and minimally invasive procedures, and aligners represent the most requested technique. Recovering impacted teeth by means of dental extrusion can definitely be a challenging movement to achieve using aligners alone. The combined use of elastics and aligners allows effective dental extrusion if the space within aligners is previously planned. Based on this concept, we can also achieve the extrusion of impacted canines if these teeth can accessibly be hooked by using elastics. Last but not least, proper ortho‐surgical planning is essential to allow the orthodontist to successfully treat impacted canines with aligners.

## AUTHOR CONTRIBUTIONS

Prof. Gianluca Mampieri, managed clinical treatment, collected clinical data and picture and wrote the manuscript. Prof. Tommaso Castroflorio, has been involved in drafting the manuscript. Dr. Andrea Conigliaro, has contributed to the references research. Prof. Aldo Giancotti, has given final approval of the version to be published.

## ETHICAL APPROVAL

For a case report, it is not necessary approval from the ethical board.

Case series and reports, being a retrospective description of clinical findings in cases or an observed course of events that document a new aspect of patient management during the normal course of clinical treatment with no hypothesis testing and no systematic data collection beyond that which is part of routine clinical practice, no data analysis, and the work has already been done do not usually qualify as "research" requiring approval from ethical boards designed to protect humans involved in clinical research.

## Data Availability

Data openly available in a public repository that issues datasets with DOIs.
